# Supplementary material for: Indomethacin reproducibly induces metamorphosis in Cassiopea xamachana scyphistomae
Source: PeerJ. 2017 Mar 1;5:e2979. doi: 10.7717/peerj.2979 (PMC5335687; doi:10.7717/peerj.2979)
Supplement: Supplemental Information 1 — Strobilation was monitored throughout 10 d of treatment at 25 °C under photoperiod (12 h light/12 h dark). Scyphistomae were mantained at 25 °C for several months before the treatment. For each replicate 3–5 scyphistomae were used; 3 replicates per treatment. (−) Indicates no changes were detected. (∼) Indicates that signs of strobilation were detected. (+) Indicates strobilation ocurred. (*) Indicates inducer concentrations at which adverse effects on the polyps were observed. FSW, Filtered Sea Water; ASW, Artificial Sea Water. [file peerj-05-2979-s001.docx]

| TREATMENT |  | DAY 1 | | | DAY 2 | | | DAY 3 | | | DAY 4 | | | DAY 5 | | | DAY 6 | | | DAY 7 | | | DAY 8 | | | DAY 9 | | | DAY 10 | | |
| --- | --- | --- | --- | --- | --- | --- | --- | --- | --- | --- | --- | --- | --- | --- | --- | --- | --- | --- | --- | --- | --- | --- | --- | --- | --- | --- | --- | --- | --- | --- | --- |
|  | REPLICATE | 1 | 2 | 3 | 1 | 2 | 3 | 1 | 2 | 3 | 1 | 2 | 3 | 1 | 2 | 3 | 1 | 2 | 3 | 1 | 2 | 3 | 1 | 2 | 3 | 1 | 2 | 3 | 1 | 2 | 3 |
| GLUCOSE | FSW | - | - | - | - | - | - | - | - | - | - | - | - | - | - | - | - | - | - | - | - | - | - | - | - | - | - | - | - | - | - |
|  | 100 µM | - | - | - | - | - | - | - | - | - | - | - | - | - | - | - | - | - | - | - | - | - | - | - | - | - | - | - | - | - | - |
| GLYCINE | 100 µM | - | - | - | - | - | - | - | - | - | - | - | - | - | - | - | - | - | - | - | - | - | - | - | - | - | - | - | - | - | - |
| GLYCEROL | 0.01 % | - | - | - | - | - | - | - | - | - | - | - | - | - | - | - | - | - | - | - | - | - | - | - | - | - | - | - | - | - | - |
|  | 0.05 % | - | - | - | - | - | - | - | - | - | - | - | - | - | - | - | - | - | - | - | - | - | - | - | - | - | - | - | - | - | - |
| GLUCOSE+ GLYCINE | 100 µM each | - | - | - | - | - | - | - | - | - | - | - | - | - | - | - | - | - | - | - | - | - | - | - | - | - | - | - | - | - | - |
| GLUCOSE+ GLYCINE+GLYCEROL | 100 µM+100 µM+0.01% | - | - | - | - | - | - | - | - | - | - | - | - | - | - | - | - | - | - | - | - | - | - | - | - | - | - | - | - | - | - |
| NITRATE | 0.8 mM | - | - | - | - | - | - | - | - | - | - | - | - | - | - | - | - | - | - | - | - | - | - | - | - | - | - | - | - | - | - |
| H_2_O_2_ | 1 nM | - | - | - | - | - | - | - | - | - | - | - | - | - | - | - | - | - | - | - | - | - | - | - | - | - | - | - | - | - | - |
|  | 10 nM | - | - | - | - | - | - | - | - | - | - | - | - | - | - | - | - | - | - | - | - | - | - | - | - | - | - | - | - | - | - |
|  | 100 nM | - | - | - | - | - | - | - | - | - | - | - | - | - | - | - | - | - | - | - | - | - | - | - | - | - | - | - | - | - | - |
| POTASIUM IODIDE | 0.341 µM | - | - | - | - | - | - | - | - | - | - | - | - | - | - | - | - | - | - | - | - | - | - | - | - | - | - | - | - | - | - |
| SODIUM IODIDE | 100 µM | - | - | - | - | - | - | - | - | - | - | - | - | - | - | - | - | - | - | - | - | - | - | - | - | - | - | - | - | - | - |
| IODIDE | 50 µM | - | - | - | - | - | - | - | - | - | - | - | - | - | - | - | - | - | - | - | - | - | - | - | - | - | - | - | - | - | - |
|  | 100 µM | - | - | - | - | - | - | - | - | - | - | - | - | - | - | - | - | - | - | - | - | - | - | - | - | - | - | - | - | - | - |
|  | 300 µM* | - | - | - | - | - | - | - | - | - | - | - | - | - | - | - | - | - | - | - | - | - | - | - | - | - | - | - | - | - | - |
| LUGOL | 130 µM / µL iodide | - | - | - | - | - | - | - | - | - | - | - | - | - | - | - | - | - | - | - | - | - | - | - | - | - | - | - | - | - | - |
| IAA | FSW+ETANOL | - | - | - | - | - | - | - | - | - | - | - | - | - | - | - | - | - | - | - | - | - | - | - | - | - | - | - | - | - | - |
|  | 50 µM | - | - | - | - | - | - | - | - | - | - | - | - | - | - | - | - | - | - | - | - | - | - | - | - | - | - | - | - | - | - |
|  | 100 µM | - | - | - | - | - | - | - | - | - | - | - | - | - | - | - | - | - | - | - | - | - | - | - | - | - | - | - | - | - | - |
| L-TYROSINE | 50 µM | - | - | - | - | - | - | - | - | - | - | - | - | - | - | - | - | - | - | - | - | - | - | - | - | - | - | - | - | - | - |
|  | 100 µM | - | - | - | - | - | - | - | - | - | - | - | - | - | - | - | - | - | - | - | - | - | - | - | - | - | - | - | - | - | - |
|  | 300 µM | - | - | - | - | - | - | - | - | - | - | - | - | - | - | - | - | - | - | - | - | - | - | - | - | - | - | - | - | - | - |
| L-TYROSINE+SODIUM IODIDE | 50 µM each | - | - | - | - | - | - | - | - | - | - | - | - | - | - | - | - | - | - | - | - | - | - | - | - | - | - | - | - | - | - |
|  | 100 µM each | - | - | - | - | - | - | - | - | - | - | - | - | - | - | - | - | - | - | - | - | - | - | - | - | - | - | - | - | - | - |
|  | 300 µM each | - | - | - | - | - | - | - | - | - | - | - | - | - | - | - | - | - | - | - | - | - | - | - | - | - | - | - | - | - | - |
| THYROXIN | FSW | - | - | - | - | - | - | - | - | - | - | - | - | - | - | - | - | - | - | - | - | - | - | - | - | - | - | - | - | - | - |
|  | FSW+DMSO | - | - | - | - | - | - | - | - | - | - | - | - | - | - | - | - | - | - | - | - | - | - | - | - | - | - | - | - | - | - |
|  | 20 µM | - | - | - | - | - | - | - | - | - | - | - | - | - | - | - | - | - | - | - | - | - | - | - | - | - | - | - | - | - | - |
|  | 60 µM | - | - | - | - | - | - | - | - | - | - | - | - | - | - | - | - | - | - | - | - | - | - | - | - | - | - | - | - | - | - |
|  | 100 µM | - | - | - | - | - | - | - | - | - | - | - | - | - | - | - | - | - | - | - | - | - | - | - | - | - | - | - | ~ | ~ | ~ |
| 9-*CIS*-RETINOIC ACID | ASW | - | - | - | - | - | - | - | - | - | - | - | - | - | - | - | - | - | - | - | - | - | - | - | - | - | - | - | - | - | - |
|  | ASW+DMSO | - | - | - | - | - | - | - | - | - | - | - | - | - | - | - | - | - | - | - | - | - | - | - | - | - | - | - | - | - | - |
|  | 1 µM | - | - | - | - | - | - | - | - | - | - | - | - | - | - | - | - | - | - | - | - | - | ~ | - | - | ~ | - | - | ~ | - | - |
| RETINOL | ASW | - | - | - | - | - | - | - | - | - | - | - | - | - | - | - | - | - | - | - | - | - | - | - | - | - | - | - | - | - | - |
|  | 0.5 µM | - | - | - | - | - | - | - | - | - | - | - | - | - | - | - | - | - | - | - | - | - | - | - | - | - | - | - | - | - | - |
|  | 1 µM | - | - | - | - | - | - | - | - | - | - | - | - | - | - | - | - | - | - | - | - | - | - | - | - | - | - | - | - | - | - |
|  | 5 µM | - | - | - | - | - | - | - | - | - | - | - | - | - | - | - | - | - | - | - | - | - | - | - | - | - | - | - | - | - | - |
| INDOMETHACiN | FSW | - | - | - | - | - | - | - | - | - | - | - | - | - | - | - | - | - | - | - | - | - | - | - | - | - | - | - | - | - | - |
|  | FSW+DMSO | - | - | - | - | - | - | - | - | - | - | - | - | - | - | - | - | - | - | - | - | - | - | - | - | - | - | - | - | - | - |
|  | 0.5 µM | - | - | - | - | - | - | - | - | - | - | - | - | - | - | - | - | - | - | - | - | - | - | - | - | - | - | - | ~ | ~ | ~ |
|  | 10 µM | - | - | - | - | - | - | - | - | - | - | - | - | - | - | - | - | - | - | ~ | ~ | ~ | ~ | ~ | ~ | + | ~ | + | + | ~ | + |
|  | 20 µM | - | - | - | - | - | - | - | - | - | - | - | - | - | - | - | - | - | - | ~ | ~ | ~ | + | ~ | + | + | ~ | + | + | ~ | + |
|  | 50 µM | - | - | - | - | - | - | - | - | - | - | - | - | ~ | ~ | ~ | ~ | ~ | ~ | ~ | ~ | ~ | ~ | ~ | ~ | + | + | + | + | + | + |
|  | 100 µM* | - | - | - | - | - | - | - | - | - | - | - | - | ~ | ~ | ~ | ~ | ~ | ~ | ~ | ~ | ~ | ~ | ~ | ~ | + | + | + | + | + | + |
